# Critical events at critical times? A gendered identity approach on the path to (sustainable) leadership

**DOI:** 10.3389/fpsyg.2022.932998

**Published:** 2023-01-04

**Authors:** Jamie L. Gloor, Stephanie K. Rehbock, Ronit Kark

**Affiliations:** ^1^School of Management, University of St. Gallen, St. Gallen, Switzerland; ^2^Talent and Organization, Accenture Strategy and Consulting, Munich, Germany; ^3^Department of Psychology, Bar-Ilan University, Ramat Gan, Israel; ^4^Business School, Exeter University, Exeter, United Kingdom

**Keywords:** gender, shocks, sustainability, identifying, work-family, work-life

## Abstract

The early career phase is a key period of identity maintenance and change. But, it is also ripe with important, attention-grabbing occurrences (i.e., critical events) that may modify these processes, particularly influencing women’s leadership pursuit. Because previous research has overlooked if or how such events might alter identifying or if these processes differ for people who identify as men and women, we integrate the identity and critical events literatures to elaborate on how positive and negative critical events may shape men and women’s identifying in the work- and non-work domains over time. We propose that critical events’ effects on identity salience will occur both within and across domains, but that these effects will be stronger within (vs. across) domains. While both positive and negative events can exert negative effects on subsequent identity salience, we propose that the effects of critical events on identity salience may be stronger for women (vs. men). Finally, we connect work identity salience with subsequent leadership status, including contextual moderators that enhance or undermine these effects (i.e., inclusive organizational climate and mega-threats, respectively). We conclude with theoretical and practical implications of this research, including for workforce efficiency and social sustainability. We also highlight calls for future research stemming from our review [e.g., sustainability critical events and gendered analyses for (more) accurate science] as well as fruitful research areas and innovative practices at the work-non-work interface for professionals on the path to leadership.

## 1. Introduction

The early career phase comprises critical, time-sensitive periods of career development (Ibarra, 1999; Modestino et al., 2019), and family formation (Grandey et al., 2020; Little and Masterson, 2022). This dynamic period is further shaped by critical events such as receiving a promotion/an award or getting married, which meaningfully affect early career professionals’ identity construction, resilience, and career success (Ibarra, 1999; Blokker et al., 2019; Kraimer et al., 2019). Critical events are important and attention-grabbing occurrences that trigger appraisal, deliberation, and (sometimes) change (Morgeson and DeRue, 2006; Crawford et al., 2019)^1^; they are highly subjective and can originate in the work- or non-work realm^2^ with positive or negative valence. While emerging evidence suggests that critical events shape individuals’ life experiences, and thus, can also trigger dynamic identity processes that inform people’s conceptions of “who they are” (Ladge et al., 2012; Ladge and Greenberg, 2015; Crawford et al., 2019), we lack a comprehensive overview of identity-based processes triggered by critical events during the early career phase.

The critical events literature (Bright et al., 2005, 2009; Seibert et al., 2013; Blokker et al., 2019; Kraimer et al., 2019) is often grounded in stress (i.e., job demands-resources perspective; Bakker and Demerouti, 2007) and motivation theories (i.e., career self-management; Deci and Ryan, 2000). While scholars argue that identity is important to study in its own right (Haslam and Reicher, 2006; van Dick and Haslam, 2012), identity processes also predict concrete career attitudes, choices, behaviors, and outcomes (e.g., job satisfaction, stress and well-being, work effort, promotions, and leadership pursuit; Lobel and Clair, 1992; Bagger et al., 2008; van Dick and Haslam, 2012; Zheng et al., 2021) above and beyond other mechanisms that have received more attention in the literature (e.g., stress, motivation, and/or resources; Deci and Ryan, 2000; Bakker and Demerouti, 2007). In other words, identity-related processes might explain a more modest slice of the explanatory pie in an empirical sense. Yet, we argue that they nevertheless represent an independent explanatory mechanism in a theoretical sense. So, by accounting for these identity-based processes, we aim to provide a more complete understanding of early career employees’ paths to leadership.

In doing so, we also explicitly integrate research on gender^3^ and critical events. Specifically, we theorize how patterns of identifying differ for people who identify as men and women. Although gender is one of the most significant and sizeable predictors of career outcomes and success (Frear et al., 2019; Zacher et al., 2019; Catalyst, 2020), existing research tends to group men and women together when discussing and analyzing critical events and their effects (e.g., Seibert et al., 2013; Kraimer et al., 2019; Akkermans et al., 2020). According to identity theory, gender is an ever-present, highly visible, and salient identity, modifying and interacting with other identities (Brewer and Lui, 1989; Stangor et al., 1992; Ridgeway and Smith-Lovin, 1999). Yet, much of the research on identity and role transitions–one type of critical event–is qualitative and focuses on only women (e.g., Ladge et al., 2012; Ladge and Greenberg, 2015; Meister et al., 2017) *or* men (e.g., Humberd et al., 2015; Ladge and Greenberg, 2015). Acknowledging the persistent and pervasive gender roles, stereotypes, and social expectations that may modify critical events’ effects for men and women—even more so for younger professionals (Eagly, 1987; Eagly and Wood, 2012; Eagly et al., 2020)—we bridge these literatures by including both men and women in our theory building while also proposing if and how critical events’ effects on identity salience may differ for early career men and women.

Finally, beyond the individual-level, we also consider two contextual moderators which affect the magnitude of the effect of work identity salience on downstream employment outcomes (e.g., future leadership status): inclusive organizational climate (i.e., organizational cultures that value their members, include them in decision-making, and treat them fairly—regardless of their social group membership; Shore et al., 2011) and mega-threats (i.e., negative, identity-relevant societal events that receive significant media attention; Leigh and Melwani, 2019). We theorize that the former strengthens the positive effect of work identity salience on leadership status while the latter undermines it. With this multi-level approach, we more completely consider employees’ everyday realities in context while also opening up new avenues for theory and practice beyond single employees. While individual approaches are indisputably valuable for understanding some phenomena and processes, they can too easily overlook the practices, organizations, and systems within which these individuals function; in doing so, they also (implicitly) place the onus on individuals to improve their situations (i.e., a “fix the woman” approach; Ely and Meyerson, 2000) despite many factors being entirely out of their control.

**Figure 1:**
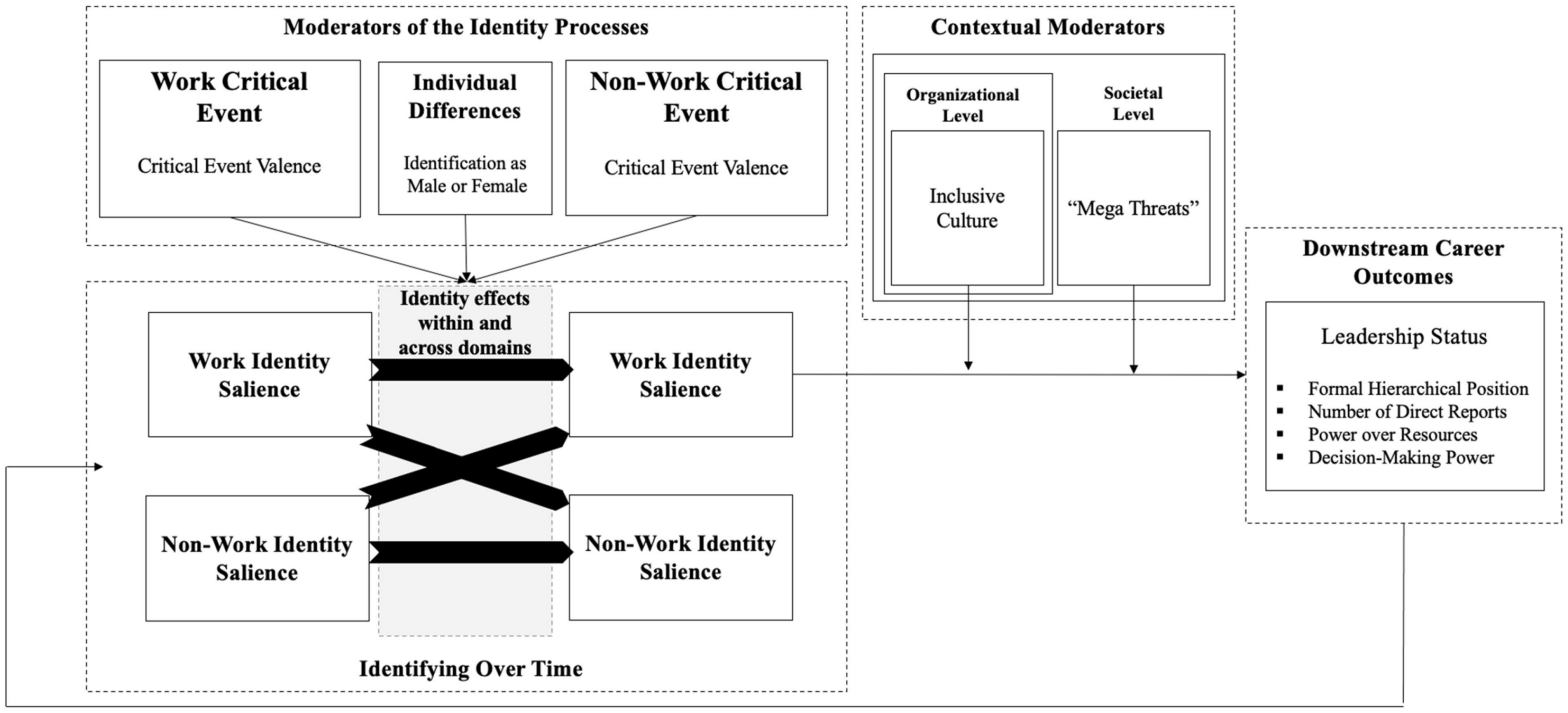
Overview of complete conceptual model. The block arrows represent processes.

In summary, this theoretical framework provides a more holistic understanding of how critical events shape early career men’s and women’s (future) leadership *via* their dynamic effects on identity salience within and across the work and non-work domains. For a complete overview of our theoretical model, see Figure 1.

## 2. Theoretical development

### 2.1. Critical events’ effects on identifying within and across life domains

Here, we explain how critical events shape early career professionals’ identifying (an ongoing process of identity maintenance and change; Sugiyama et al., 2022), particularly for a specific aspect of identity, namely: identity salience. People possess multiple identities which differ in salience. “Identity salience is conceptualized (and operationalized) as the likelihood that the identity will be invoked in diverse situations” (Hogg et al., 1995, 257). The more salient an identity, the more likely it is to be evoked in a social interaction (Brenner et al., 2014). According to various identity theories (e.g., Stryker and Serpe, 1994; Stryker and Burke, 2000; Epitropaki et al., 2017), people implicitly arrange their identities into salience hierarchies, with more highly salient identities more likely to be deemed situationally relevant and subjectively important (McCall and Simmons, 1978; Ashforth, 2000). Thus, because critical events or “shocks” may be often experienced and trigger important identity processes (see Ibarra and Barbulescu, 2010; Ibarra and Petriglieri, 2010; Crawford et al., 2019) during this dynamic, uncertain early life, and career stage, it is important to understand how critical events shape young women’s and men’s identity salience.

Despite the more dynamic quality of identity as people grow and develop over the lifespan (Ibarra, 1999; Sveningsson and Alvesson, 2003; Kreiner et al., 2006), people generally maintain a sense of identity continuity to behave effectively (Shamir et al., 1993; Ashforth and Kreiner, 1999; Petriglieri, 2011). That is, initial work identity salience at one point in time should be strongly and positively related to subsequent work identity salience, and non-work identity salience at one point in time should also be strongly and positively related to subsequent non-work identity salience. When critical events occur in the work or non-work domain, it is highly likely that they affect identity salience stronger in the domain in which they occur. For example, if a young woman gives birth or has a miscarriage, the effects of this critical event in the non-work domain may be most noticeable in her non-work identity salience. Similarly, if a young man is fired (or promoted) from his work, the effects of this critical event in the work domain may be most noticeable in his work identity salience. Because shocks research also supports the idea of valence-consistent effects within domains (e.g., Seibert et al., 2013; Blokker et al., 2019; Kraimer et al., 2019), we similarly propose that critical events have stronger effects within its domain of origin.

But, the work–family literature also shows interrelated aspects of work- and non-work-related self-concepts, which may have counterbalancing *or* enhancement effects on identity in the other domain (Wayne et al., 2006; Ladge and Little, 2019). Much research supports the former idea, such that individuals’ roles and responsibilities within one domain exert a compensatory effect on identity and activities in the other domain (e.g., Bagger et al., 2008; see Greenhaus and Beutell, 1985, for a review), which we refer to as a cross-domain effect. This idea is consistent with the depletion perspective (see Edwards and Rothbard, 2000, for a review; Rothbard, 2001)—a fundamental aspect of work life theories—reflecting the idea that from a fixed pool of resources (e.g., time and energy), engagement in one area reduces the resources available in another area (Lambert, 1990).

While identity is not necessarily a resource, identity salience hierarchies are necessarily structured along subjective importance (McCall and Simmons, 1978; Stryker, 1987; Ashforth, 2000). This implies a trade-off between various sources of identity salience. Indeed, following the “hat” metaphor by Ashforth and Johnson (2001) to describe the relative salience of multiple identities in organizational contexts, one person cannot truly wear “two hats” at the same time. While we do not intend to singularly promote a zero-sum approach to all theorizing on cross-domain effects, at least for identity salience, related empirical research suggests that compensatory effects may be more likely than enrichment effects (e.g., Lobel and Clair, 1992; Bagger et al., 2008). However, admittedly, there is only a paucity of work-family research on cross-domain identity processes in response to critical events.

Thus, we propose that through identifying, a critical event may have manifold effects on identity salience beyond its initial domain of origin to cross-over and impact multiple domains (e.g., work and non-work). We further predict that the effects of an individual’s critical event—in the work or non-work domain—resonate more strongly in the domain in which it originated, shaping identity salience more prominently in that domain than potential cross-domain effects.

*Propositions 1a-b: Critical events affect early career professionals’ work and non-work identity salience, particularly* (*a*) *within the domain of its origin* versus (*b*) *across domains*.

### 2.2. Critical event valence and identity effects

Critical events can be positive or negative in valence. Existing research has shown that critical events tend to have valence-consistent effects within their domain of origin. For example, a promotion is an ostensibly positive critical career event associated with positive career outcomes (Seibert et al., 2013; Blokker et al., 2019; Kraimer et al., 2019). Although these studies were guided by stress or resource frameworks, meaning that positive events triggered their positive effects because they decreased stress or increased resources (respectively), similar claims could also be made based on identity theory for events within domains. To illustrate, a positive critical event in the work domain (e.g., an assignment abroad to gain essential international experience and climb the corporate ladder) may invoke a leaders’ work identity, requiring investment in the work role and identity (Crawford et al., 2019; Kraimer et al., 2019), and thus, increases work identity salience. But, cross-domain effects may also occur with an opposite pattern of less magnitude. More specifically, by increasing identity salience in one domain, other aspects of identity become inherently less salient, decreasing in subjective importance (McCall and Simmons, 1978; Ashforth, 2020).

At first glance, negative events may be logically expected to trigger negative effects. For example, if one experiences a major setback at work, they may respond by reducing their work identity salience (and also their work effort, etc.). But in contrast to the valence-consistent effects of positive critical events, negative critical events may also cloud or obscure identity consistency over time, resulting in more variable responses on subsequent identity salience. For example, in a related study of shocks, Blokker et al. (2019) found that positive career shocks strengthened the relation between career skills and outcomes, while negative career shocks undermined this relation. This may be especially likely for early career individuals (Miller et al., 2005) who may reconsider or postpone having children or taking on a mortgage to prevent having “one more worry” during a difficult period (e.g., see Akkermans et al., 2020). Hence, a negative critical event can strengthen identity salience in some cases (e.g., losing one’s job may enhance family engagement), but with a broader outlook, they may simply reduce the strength of identity salience within or across domains over time.

This theorizing is also supported by the limited research on critical events and shocks that has considered the role of event valence. Although this work tends to focus on positive *or* negative events (e.g., Seibert et al., 2013) or propose specific effects of critical events regardless of event valence (e.g., Crawford et al., 2019), existing research that has considered both types of critical events shows more consistent empirical support for the valence-consistent effects of positive shocks than negative shocks (e.g., Blokker et al., 2019; Kraimer et al., 2019). Related work on leader identity development also suggests positive events strengthen existing identity salience and identifying processes more so than negative events (e.g., Seemiller and Priest, 2015; Epitropaki et al., 2017).

In summary, we propose that positive events enhance positive, within-domain identity effects as well as the negative, counterbalancing effects across-domains. In contrast, we propose that negative events may generally decrease both effects. The idea that positive and negative events may affect not only the direction but also the magnitude of subsequent effects is supported by theory on critical events (e.g., Morgeson et al., 2015) and empirical research on shocks and chance events (e.g., see Grimland et al., 2012; Seibert et al., 2013; Blokker et al., 2019; Kraimer et al., 2019). Formally:

*Propositions 2a-c: Critical events’ effects on identity salience within and across domains depends on the valence of the events, such that* (*a*) *positive events are more likely to strengthen identity salience in the domain of origin and* (*b*) *reduce it in the cross domain* (e.g.*, a positive event in the work domain strengthens identity salience in the work domain and weakens identity salience in the non-work domain and* vice versa). *Furthermore,* (*c*) *positive* (vs. *negative*) *events should generally have stronger effects* (*within and across domains*).

### 2.3. Critical events, identity salience, and gender

Gender is a fundamental, deeply engrained, and prominent category by which we classify ourselves and others (Hentschel et al., 2019; Martin and Mason, 2022). Gender-based taxonomies emerge already in early childhood with such strength that even the multiple dimensions within one’s identity are cognitively nested within gender categories (Brewer and Lui, 1989; Stangor et al., 1992; Ridgeway and Smith-Lovin, 1999). Thus, gender is a highly visible and ever-present identity, modifying other identities which may be more salient.

Following this reasoning, the previously proposed effects of critical events on identity salience may depend on the focal employee’s gender. Chiefly important to our theorizing, women may be more sensitive to context than men in their identity formation as well as in their reactions to critical events within those contexts, because they are stereotyped as a minority (e.g., in career roles or at work) and/or they are a numerical minority within the workplace and public sphere domain (Randel, 2002; Gloor et al., 2020). Evidence from identity research supports this idea, as women leaders in male-dominated fields are more strongly impacted by professional and personal identity transitions (Epitropaki et al., 2017; Meister et al., 2017). Because women are more scrutinized while also having to address multiple and paradoxical expectations (Kark et al., 2012; Meeussen et al., 2016; Zheng et al., 2018a,b, 2021), they may be more vulnerable than men, which may translate to stronger effects of critical events on identity salience for women.

A related stream of work-family research shows that men and women have different work-life boundary strength or permeability (Rothbard and Edwards, 2003). According to boundary theory, individuals construct psychological boundaries between different domains in their lives (e.g., work and private life) while also acknowledging that boundaries vary in permeability, namely, the degree to which one domain can influence the other (Ashforth et al., 2000; Kossek et al., 2012; Leslie et al., 2019). These work-family boundaries have been described as more fluid and permeable for women than for men, because of women’s relatively stronger need to integrate work and family roles (Rothbard and Edwards, 2003; Halpern and Murphy, 2005; Cheung and Halpern, 2010; Powell and Greenhaus, 2010; Brown, 2015; Braun and Peus, 2018). This suggests that women’s work-family boundaries are also likely to be more permeable than men’s boundaries. More specifically, women may more strongly identify with both the work and non-work domains, whereas men may relate more to the work domain while also overlooking the need to integrate both domains.

Thus, we theorize that the previously formulated effects of critical events on identity salience both within and across domains may also be stronger for women than for men. Formally:

*Proposition 3: Critical events’ effects on identity salience will be moderated by gender, such that the effects are stronger for early career professionals who identify as women* (vs. *men*).

### 2.4. Work identity salience and leadership

While identity is an important outcome worthy of study on its own (Haslam and Reicher, 2006; van Dick and Haslam, 2012), aspects of employee identity also predict concrete career attitudes, behaviors, and outcomes (e.g., job satisfaction, stress and well-being, work effort, promotions, and leadership pursuit; Lobel and Clair, 1992; Bagger et al., 2008; van Dick and Haslam, 2012; Zheng et al., 2021). Specifically, the identity literature focuses more on internalized perceptions that build the basis for behavior (see Haslam and Reicher, 2006). So, if a person has a strong work identity salience, they will also behave accordingly to prioritize job-related tasks over others, seek professional development and career opportunities, etc. The leadership literature has highlighted that identity motivates behavior in that professionals who identify as a leader will be motivated to take on leadership responsibilities and search for opportunities to further develop in that direction (Lord and Hall, 2005; Rehbock et al., 2022). Due to this strong link between identity salience and behavioral enactment (Strauss et al., 2012), building theory with an identity lens seems fruitful to enhance our understanding of how changes in work identity salience shape young professionals’ work behavior.^4^

To illustrate, if an early career employee experiences a critical negative event in the work domain (e.g., an incident with an abusive boss or an act of harassment), it likely weakens their work identity salience, undermining subsequent leadership outcomes and steps along the way to leadership (e.g., a weakened motivation to lead and/or ambition to apply for more senior projects/roles). Alternatively, if an early career employee experiences a positive critical event in the work domain, such as winning a valuable prize or receiving an early promotion, it likely enhances their work-identity salience, strengthening subsequent leadership outcomes and steps along the way to leadership (e.g., a greater motivation to lead and/or ambition to apply for more senior projects/roles).

Thus, we focus on how work identity salience predicts subsequent work outcomes related to leadership. While not all employees strive for leadership roles, we have at least implicitly focused our theorizing on early career professionals who have at least some initial leadership ambitions until now, a point that we now aim to make explicit. So, to be clear: while years of time may pass before employees achieve various leadership statuses—and it can also take on various forms (e.g., more direct reports, more power in terms of control over resources and/or decision-making tasks, a position that is formally higher in the hierarchy, etc.; see Figure 1); we keep it intentionally broad here to include related leadership roles, tasks, and leadership responsibilities. Formally:

*Proposition 4: Early career professionals’ stronger work identity salience positively predicts subsequent leadership status*.

### 2.5. Contextual moderators

Finally, there are also broader elements beyond individuals which may influence if or how professionals’ work identity salience affects their subsequent leadership. While non-work identity salience could also be theoretically related to subsequent leadership, within-domain effects tend to be stronger and more consistent (e.g., work- or career-identity salience predict work- or career outcomes; Lobel and Clair, 1992), so we focus again on work-identity salience as in Proposition 4.

We review two key contextual elements here: inclusive culture (i.e., organizational cultures that value their members, include them in decision-making, and treat them fairly—regardless of their social group membership; Shore et al., 2011) and mega-threats (i.e., societal-level critical events, which receive media attention, are negative in valence and identity-relevant; Leigh and Melwani, 2019). Inclusive culture and mega threats are situated at broader levels compared to most of the previously reviewed critical events, which are largely situated at the individual level. Because such higher-level critical events may entail more frequent cues (e.g., more people are involved in or affected by the events, more media coverage of the events, etc.), this makes these contextual moderators qualitatively different from the previously reviewed individual-level critical events, necessitating a new part of our model and conceptual space in our theory-building.

As a first contextual moderator, we integrate recent theorizing on (gendered) identity sensemaking and leadership “imposterism” from Kark et al. (2022) to propose that inclusive organizational climates affect the positive relation between work identity salience and (future) leadership for three reasons. First, in more inclusive organizational climates, demographic factors (e.g., gender, age, motherhood, race, etc.) are not as strongly related to status, facilitating employee evaluations which are more indicative of their ability and potential rather than their visible characteristics (DiTomaso et al., 2007; Nishii, 2013). Thus, inclusive climates may reduce the extent to which those who differ from the societal prototype of leaders (e.g., in terms of gender, age, motherhood, race, etc.) feel that their identity is misaligned with their desired career role (e.g., leadership). Second, inclusive organizational climates are less likely to trigger identity-related sensemaking processes among (future) leaders, because they encourage greater interdependence and mutuality (Ferdman, 2014). Unlike more traditional, highly hierarchical organizations that expect individual, “hero” employees to have all of the answers as they climb the organizational hierarchy (Hollander, 2009); inclusive climates place less pressure on individuals. By definition, inclusion comprises being fully oneself while also allowing others to be fully themselves in the context of engaging in common pursuits. Thus, collaborating is a way that all parties can be fully engaged, and yet at the same time, paradoxically believe that they have not compromised, hidden, or given up parts of themselves in the process. Finally, some organizational initiatives and policies (e.g., if important meetings and events are held in a common language—or perhaps multiple languages, as needed—and within versus after typical work hours, childcare and parental leave offerings, etc.; Gloor et al., 2021a) can also be key signals of organizational inclusion—as well as organizational responses to patterns of organizational exclusion (e.g., higher collective female turnover; Piszczek, 2020).

As a second contextual moderator, we integrate recent theorizing on mega-threats from Leigh and Melwani (2019, 2022) to propose that mega-threats affect the positive relation between work identity salience and (future) leadership for three reasons. Recent years have been peppered with mega-threats at the broader societal level which have undeniable effects on organizations and the people whom they employ. For example, COVID-19 could be a mega-threat for people of Asian descent (because it triggered harassment and aggression toward people of apparent Asian decent), while police killings of people of color could be a mega-threat for people of color (Leigh and Melwani, 2022). Similarly, the recent #MeToo movement (see Gloor et al., 2022b) and the very recent unraveling of women’s reproductive rights in the United States (Thomason et al., 2022) might constitute mega threats for women—the latter particularly for women of childbearing age and those who may want (more) children. Of note, mega-threats are negatively valenced by definition, in contrast to the subjectively positive and negative critical events that we focused on previously (e.g., in Proposition 2). Mega-threats like these may play a crucial role in how work identity salience impacts downstream outcomes like a future leadership role, because they increase avoidance behaviors, increase work withdrawal, and decrease social engagement in event observers who share identities with mega-threat victims (germane to the current research, these observers include early career professionals who share identities with mega-threat victims; Leigh and Melwani, 2022). Mega-threats theoretically enact these effects because they blur work- and non-work identity boundaries (Leigh and Melwani, 2019)—which we previously argued is a reason why women may be more affected by critical events than men (see Proposition #3)—while also trigging vicarious harm and embodied threat (i.e., an appraisal that one is more likely to personally encounter identity-based harm; Leigh and Melwani, 2022). For each of these examples, they are also broader, societal—if not global—events that foster discussions at work, affect multiple individuals with whom one might interact with at work, while also generating widely shared media attention. For these reasons, mega-threats may also have (more) frequent cues.

Thus, more inclusive organizational climate can reduce the negative (and enhance the positive) identifying processes resulting from more individual critical events predicting subsequent leadership status. In contrast, mega-threat(s) can exacerbate the negative (and undermine the positive) identifying processes resulting from more individual critical events predicting subsequent leadership status. But while this theorizing explains how these two contextual moderators shape the dynamic identifying processes proposed in the first stage of our model, we focus our theorizing here more specifically on how these contextual factors affect the (positive) relation between work identity salience and subsequent leadership status. In doing so, we more centrally build on Proposition 4 to further theorize how these two contextual factors may individually affect the baseline positive relation between work identity salience and downstream outcomes like leadership status.^5^ Formally:

*Propositions 5a-b: Contextual factors moderate the positive relation between work identity salience and subsequent leadership status, such that* (*a*) *inclusive organizational climates strengthen this relation, while* (*b*) *mega-threats weaken this relation*.

In summary, we propose that critical events can shape identity salience both within and across domains, but that they trigger stronger effects within (vs. across) domains. While positive events may strengthen positive, within-domain identity effects and the negative, cross-domain effects, negative events may weaken both effects. Furthermore, we propose that these effects are stronger for people identifying as women than for people identifying as men, because women are more sensitive to context and have more permeable work-family boundaries than men, which means that women may react more strongly to critical events than men. We then connect identity salience to important downstream outcomes such that work identity salience positively predicts early career professionals’ (future) leadership status. Finally, we also consider contextual moderators that shape the magnitude of the positive relation between work identity salience and leadership status—inclusive organizational climate and mega-threats: while the former enhances this effect, the latter undermines it.

## 3. Discussion

Integrating the critical events and gender/diversity literatures with an identity lens, we explored the idea that positive and negative critical events shape early career professionals’ identity salience, particularly within—vs. across—domains, generally triggering stronger effects for women than for men. While we theorized that work identity salience predicts downstream outcomes like leadership, the downstream effects of these dynamic identifying processes in response to critical events are moderated by key aspects of the context: how inclusive the organization is and the presence of mega-threats. Next, we discuss the implications of our model for theory and practice.

### 3.1. Theoretical implications

First and foremost, we built theory about how critical events affect identifying over time. In doing so, we could more accurately predict and outline the effects of positive and negative critical events and their effects on employees’ identity salience. This builds on prior literature that has treated work and non-work (often family) identities as separate (e.g., Greenhaus, 1971, 1973; Amatea et al., 1986; Lobel and Clair, 1992; Bagger et al., 2008). Instead, and in line with more recent theorizing on identity processes at the work-family interface (e.g., Crawford et al., 2019; Ladge and Little, 2019), we theorized that work- and non-work (family) identity salience likely enjoy a process of co-evolution through cross-domain effects, particularly in the wake of positive events.

Second, we also conceptually explored the idea that the effects of critical events are stronger for women than for men. In doing so, we aim to extend existing knowledge of critical events and shocks which has grouped employees together to analyze the effects of critical events (e.g., Seibert et al., 2013; Blokker et al., 2019; Kraimer et al., 2019). By considering gender as a primary identity component and a major aspect of the process through which critical events affect work- and non-work-related outcomes, we may better understand if and how early career men and women respond to critical events. In doing so, this research also aims to complement research in related areas (i.e., critical events and identity transitions), which tends to focus on men *or* women (e.g., Ibarra, 1999; Ladge et al., 2012; Ladge and Greenberg, 2015; Meister et al., 2017; Crawford et al., 2019).

We theorized that women are more susceptible or sensitive to critical events and their identity-related effects than men due to their relatively lower power and status in organizations (Catalyst, 2020; Henningsen et al., 2022) and because of the dual and multiple societal expectations and pressures that women may experience and internalize in earlier adult ages (Meeussen et al., 2016; as previously described). However, this idea also builds on recent theorizing on the physical, bodily changes that may also make women more vulnerable to critical life events, particularly within this early- to mid-career period (e.g., Grandey et al., 2020). Because women may be more vulnerable at work and more involved in childbearing and rearing at this stage than men (Gersick et al., 2000; Grandey et al., 2020; Little and Masterson, 2022), they are likely more attuned to or affected by critical events, many of which are related to their personal life experiences. Related research supports this idea, because women are also more field dependent than men (i.e., more reactive to external stimuli; Haaken, 1988; Martin, 2000). While this field dependency has been interpreted as a “lack of independent thinking and a regrettable inability to separate one’s reactions from contextual influences” (Calás and Smircich, 1992, p. 232–233), this “contextual sensitivity” may also be strength (e.g., see Haaken, 1988). For example, leaders who are more sensitive to context may also perform better along progressively vital social and environmental sustainability outcomes (see Matsa and Miller, 2013; Post, 2015; Byron and Post, 2016). Hence, instead of focusing on women’s sensitivity as a weakness to be overcome, it may (also) indicate a need to help men in strengthening their sensitivity to context—including, but not limited to critical events. Indeed, young men may be more influenced by new norms that prescribe men to invest more in their family, suggesting a potential opportunity for change (Meeussen et al., 2016), perhaps especially in the wake of a (positive) critical event.

Considering the greater permeability of work-life boundaries for women than for men, one could also expect gender to function as a moderator for cross-domain effects of a critical event in one domain on identity salience in the other domain. For example, getting married, a critical event in the non-work domain may have stronger effects for women’s work identity salience than for men’s work identity salience. This is because women may be more sensitive to—and more often confronted with—external expectations about their new role identity as a legal partner and/or a potential parent (see Rivera, 2017; Gloor et al., 2018a, 2021b). Similarly, the latter part of our model might also be more precisely depicted with moderation by employee gender. That is, inclusive climate and mega-threats might be more influential for women (vs. men)—just like the front-end of our model—for some of the same reasons (e.g., women are more sensitive to context and have weaker boundaries between work and non-work domains compared to men) and because women are often the target of mega-threats [e.g., the recent undermining of women’s (reproductive) health and rights in the United States]. But, many of these mega-threats are driven primarily by race/ethnicity (e.g., mass shootings, police brutality, and killings of specific groups; Leigh and Melwani, 2019, 2022)—not gender; so, while an intersectional approach may be fruitful here to explain the process and predict leadership outcomes, it becomes quickly complicated due to the multiplying number of categories (e.g., race/ethnicity, plus gender, and oftentimes age) as well as diverging predictions (e.g., for Asian women vs. Black women; see Hall et al., 2019, for a particularly lucid review). Thus, while out of scope here, we encourage future research to more thoroughly explore if and how our model might be depicted (e.g., with an intersectional lens).

Finally, despite increasing research on critical events, shocks, and related concepts, our review of the literature—and thus, also our theory-building—was admittedly limited, because it focused on “typical” professional and personal events (e.g., job loss or childbearing) and largely took a human resource management perspective. Together, these factors limit our understanding of how sustainability affects gendered critical events (and vice versa), as well as the implications of these dynamics for (future) leaders—critical issues to better tackle grand challenges. For example, climate change creates social perils like conflict and extreme weather (Zhang et al., 2007), which may trigger one (or more) critical events; these events may not only differ from those we previously reviewed, but they may also trigger more critical events (see United Nations, 2018; Gloor et al., 2022a). We also know that social and environmental sustainability are deeply related, because vulnerable populations—including, but not limited to women—are more frequently and severely affected by climate change and related issues while women may also be uniquely positioned to lead towards more (social) sustainability (Byron and Post, 2016; Chang et al., 2022; Gloor et al., 2022a; Matsa and Miller, 2013).^6^ Given the short timeline to meet environmental goals, paired with widespread global talent shortages (Franzino et al., 2022) and the increasing numbers of (climate) migrants who may be particularly prepared to tackle these challenges, scholars and organizations should not overlook these “sustainability *mega*-critical-events” and their multifaceted implications for theory and practice.

### 3.2. Practical implications

One recommendation from our research for early career professionals could include active identity-based reflections. In doing so, these early career professionals may grow more aware of their valued identities in various domains, and thus, be better prepared to consciously adapt their self-views, if/when needed (see Roberts, 2005). For example, professionals can implement regular reflection sessions on a monthly or semi-annual basis by answering questions such as “Who am I as a professional?,” “What is important to me?,” “What (un-)expected events took place and what do they mean to me?,” and “How did/do critical events in the past month or year change what I want from my career and/or in my personal life?” (see also Rehbock et al., 2021, for suggestions of active identity reflections for organizational leaders). Managers and leaders can support these reflections by introducing them in regular conversations with their employees, annual meetings, etc.

Extending from our theorizing around inclusive organizational climates, leaders (e.g., group leaders, supervisors, and other leaders such as deans and heads of departments in academia) would do well to promote a culture where employees do not feel that they are alone or that they need to decide between their career *or* their personal life to succeed or climb the organizational hierarchy into a leadership role. Because supervisor support strategies often take the form of informal arrangements (Kossek et al., 2016), an open and trustworthy relationship between employees and leaders may help to meet individual employees’ needs. However, leaders can further promote inclusion and supportive, compassionate cultures toward employees in their teams (Leigh and Melwani, 2019) by showing value for and acceptance of employees during critical events—and perhaps especially in the wake of mega-threats—for example, by showing commitment to employees’ needs (Ladge and Little, 2019) and assuring psychological safety around identity-related discussions (Leigh and Melwani, 2022).

More generally—and building on our brief discussion of workplace initiatives and policies in the previous section about inclusive organizational climate—flexible work arrangements, policies, and practices at the organizational level could also enable employees to balance their multiple needs in work and family domains (Ladge and Little, 2019). To facilitate long-term success, such efforts must be career enabling–rather than career enclosing (Bourdeau et al., 2019)–and offered to *all* employees, ideally in an opt-out rather than opt-in fashion (e.g., see Gloor et al., 2018b; Erkal et al., 2022) to reduce bias and career consequences that may systematically (dis)advantage those from particular social groups. Emerging evidence also suggests that an opt-out approach (vs. the more common opt-in) may also increase qualified women’s pursuit of leadership roles (Erkal et al., 2022).

With the broader career scope in mind, and because the largest share of trained female talents is lost (or pushed out) during the early career phase on which we focused, we hope that this research might also inform the persistent and pervasive gender gaps in leadership positions across academia and organizations (e.g., Kossek et al., 2016; Catalyst, 2020; Rehbock et al., 2021; Henningsen et al., 2022). Women often leave and/or are lost after critical events and shocks like the ones we highlighted here (e.g., pregnancy; see Gloor et al., 2018a; Paustian-Underdahl et al., 2019; Zacher et al., 2019; Arena et al., 2022). However, men and women in more advanced career stages or leadership roles can proactively offer support as mentors, sponsors, and allies—by speaking openly about how to successfully integrate multiple identities from the work and non-work domain without having to choose one over the other. Increased awareness of how early career women’s and men’s identity salience and leadership pursuit differ in response to critical events may be fruitful areas for organizational allyship, thereby facilitating workforce sustainability and advancing more gender balance in representation and power where it is still particularly needed at later career stages.

Finally, some policies show promise to facilitate female retention regardless of the identity processes underway (e.g., reliable and affordable childcare provisions; Piszczek, 2020; or a simple résumé intervention to help women return to work after a caregiving leave; Kristal et al., 2022). Because biased turnover undermines workforce and economic sustainability, innovative approaches may also be fruitful here. For example, one organization successfully retained their employed female talents around a specific critical event—childbearing—by providing all pregnant women with a small, discretionary budget they could use to meet their diverse needs (e.g., hiring a research assistant to monitor data collection while on leave or paying for childcare help)—they only needed to formulate a concrete plan with their supervisor prior to childbirth (Hering, 2019). This approach is flexible to meet the diverse needs of early career female talents, delivered in an opt-out approach while also creating accountability—all of which are effective mechanisms from behavioral science (Bohnet, 2016). Thus, such innovative strategies could also help other organizations to retain early career female talents, fortifying their leadership pipeline, and increasing workforce efficiency more broadly.

### 3.3. Strengths, limitations, and future research

Two key limitations related to our theorizing are particularly worthy of note. First, conceptually, identity is a vast concept answering the question “who am I?” (Stryker and Serpe, 1982, p. 206). Here, we focused on one specific aspect of identity: salience. Thus, we encourage future research to expand beyond this singular, albeit critically important and influential, facet of identity. While identity centrality is a more stable aspect of identity which may be less affected by the context—including, but not limited to—critical events (Kreiner et al., 2015), posing challenges for theoretical and empirical work, the concept of misidentification (i.e., internal identity asymmetry; see Meister et al., 2014, 2017) might provide fruitful grounds for both types of research.

Second, critical event valence is a key factor related to the form and magnitude of effects on identity salience. While valence can be very subjective, we largely focused here on the more normative interpretations of key critical events (e.g., in Propositions 2a–c and Table 1). While we believe this event-oriented approach (a la Morgeson et al., 2015) represents a conceptual and methodological improvement by disentangling cause and effect compared to existing shocks research which tends to conflate event valence with its effects (e.g., an event is considered to be “negative” if it has a negative effect on a downstream career outcome; Seibert et al., 2013; Kraimer et al., 2019), this approach also represents an oversimplification of reality. To remedy this, we encourage future research to prospectively analyze critical event content and individuals’ subjective evaluations of event valence separately from their subsequent effects on various outcomes (e.g., identity salience, leadership status, etc.).

**Table 1:** Overview of examples of key (Early Career) critical events.

Critical events	Valence	
	Positive	Negative
Work Domain	(Early) Promotion	Passed Over for Promotion
	New Position/Employment	Contract Ending
	Further Education	Job Loss
	Award/Honor for Achievements	Act of Harassment/Discrimination
	Career Choice (Desired)	Career Change (undesired)
Work- and Non-Work Domain	(Available) Parental Leave	(Lack of/Too Much) Parental Leave
	Moving (Desired)	Moving (Undesired)
Non-Work Domain	New Relationship	Separation/Divorce
	Moving in with a Partner	Forced Removal from One’s Home
	Sabbatical/Decision to Travel	Health Issues, Accident
	Marriage	Death of a Loved One or Partner
	Pregnancy/Having a Baby	Having an Abortion/Miscarriage

The aim of this table is to provide a clear overview of some key examples of early career critical evens originating in various domains that may be normatively positive or negative in valence. However, that these events are highly subjective and not always uniformly experienced as positive or negative; we tried to explicitly account for this in some cases (e.g., parental leave can be quite positive for women’s health and recovery after having a child, as well as men’s and women’s bonding and adjusting within the family; but, it can also be quite negative for one’s workplace experience and trigger negative career penalties—particularly with longer maternity leaves; e.g., Gloor et al., 2018a; Hideg et al., 2018). However, we also explicitly acknowledge the trade-off between trying to build inclusive theory—generalizing to a broader number of events in our theorizing—while also attempting to accurately depict (the average) individual’s experience(s).

Beyond individual identity processes, structural shocks may also influence more relational and collective identity processes. For example, there are three different levels of self (Brewer and Gardner, 1996; Brickson, 2000), which are triggered by the context in ways that can affect identity salience. The personal self refers to the individual level of the self-concept, mainly focused on one’s characteristics, attributes, and self-interest; the relational self is derived from dyadic and small group relationships, as well as from the roles individuals hold in relations to others (e.g., manager, employee, etc.). Thus, this aspect of identity is mainly focused on the responsibilities and responsiveness that comes with their roles and relationships towards others’ needs. This more collective self is based on the individuals’ connections to a collective, a group or wider community (e.g., organization, state, country, etc.) and is mainly focused the obligation and commitment to the group’s welfare (Brewer and Gardner, 1996). Following this logic, different critical events and the associated identifying processes do indeed affect individual employees across these multiple levels. Thus, changes in the social structure, manifested in changes in relationships (e.g., organizational layoffs or restructuring of many colleagues), can also influence early career professionals’ identity salience. These changes can influence the relational levels, but also the collective level (e.g., if a person leaves the organization or takes a leave of absence due to some critical life event), possibly reducing early career employees’ work identity salience. Future research is needed to more thoroughly explore and test these ideas.

Similarly, men and women often have partners who also work (i.e., dual-career couples; see Crawford et al., 2019). Thus, while we focused on individual men and women in our theorizing, it is also possible that the critical events and the subsequent identity-related processes triggered by these events also affect the focal men or women’s partners’ identity salience. While new research by Little and Masterson (2022) considered the direct, indirect, and shared crossover mechanisms of specific critical events (e.g., having a child and returning to work) on organizationally-relevant outcomes grounded in resource- and stress-based theories, sensemaking processes at the partner-level may also facilitate identity-spillover effects among couples (see Crawford et al., 2019). Thus, even if critical events more strongly affect women’s identity salience, they may still meaningfully affect women’s partners (often men) and these partners’ identity salience, as well.

Finally, previous research has called for explorations of gendered effects in the context of critical events or shocks (e.g., Kraimer et al., 2019); here, we take this request one step further: at a minimum, future research should not only consider the potential main effects of gender or including it as a covariate, but scholars should also consider its potential moderating effects. In light of our Proposition 3, for example, it could be that the previously reported effects of critical events or shocks not only differ for men and women but may be entirely driven by women. If true, this is no minor issue, because social scientists’ inaccurate over-generalizations about empirical findings—even if unintentional—impedes progress in our understanding of empirical phenomena *and* social justice gains in terms of assessing and improving professional experiences and career progression for more equity in leadership positions and in organizations more broadly (see Eagly, 2016).

## 4. Conclusion

We theoretically explored the effects of critical events on early career professionals’ work- and non-work identity salience over time, including if these effects differ by event valence or for men and women. We further considered the effect of work salience on (future) leadership status, including the roles of inclusive cultures and mega-threats. Thus, this theoretical work highlights key insights for a more holistic understanding of early career professionals’ work- and non-work experiences and their identity-related effects, such that not all critical events may trigger changes over time. Instead, positive (vs. negative) critical events may trigger stronger effects on women’s (vs. men’s) identity salience within (vs. across) domains—especially in less inclusive climates and/or in the presence of mega-threats—with implications for leadership pursuit and (social) sustainability more broadly.

## Author contributions

JG, SR, and RK contributed to the conceptualization, writing—original draft, and review and editing. All authors contributed to the article and approved the submitted version.

## Funding

This research was supported by a Swiss National Science Foundation grant (PR00P1_193128) awarded to JG. However, the funder had no role in study design, data collection and analysis, decision to publish, or the preparation of the manuscript.

## Conflict of interest

SR is now employed by Talent and Organization, Accenture Strategy and Consulting.

The remaining authors declare that the research was conducted in the absence of any commercial or financial relationships that could be construed as a potential conflict of interest.

## Publisher’s note

All claims expressed in this article are solely those of the authors and do not necessarily represent those of their affiliated organizations, or those of the publisher, the editors and the reviewers. Any product that may be evaluated in this article, or claim that may be made by its manufacturer, is not guaranteed or endorsed by the publisher.

## Footnotes

^1^We generally use "critical events" (i.e., distinguishable occurrences) throughout, but we occasionally reference "setbacks" and "shocks" (i.e., "a very distinguishable event that jars employees toward deliberate judgments about their jobs"; Lee and Mitchell, 1994, p. 60), as well as "chance events" (i.e., "unplanned events"; Hirschi, 2010), where relevant. Although their definitions vary slightly, we believe the overarching concept and its potential effects on identifying, career decisions and trajectories are similar enough to justify this cross-fertilization of terms and literatures. See Table 1 for examples.

^2^Considering the most common kinds of critical events that early career professionals might experience (see Table 1), the work and non-work (often family) domains seem to be particularly relevant. To be clear, we move beyond the two-domain approach of "work" and "family" to more comprehensively and inclusively reflect other roles from which a person might derive meaning and identity. But, much of the existing, relevant research has focused on "family;" with the rise of dual career couples and the fact that the concurrent periods of family formation and early career which we focus here, much of the work we review and the examples we include still reflects "family."

^3^Here, we focus on gender as a binary construct rather than reflecting the more complex, continuous diversity in people’s gender identity (see Morgenroth and Ryan, 2018). By using "gender," we mostly relate to cis women and men, largely due to constraints from the literature we review, which used a similarly binary approach. However, here, we proactively acknowledge the continuum of gender self-definitions as well as the multiplicity of gender identities; we also acknowledge the potential limitations that our binary conceptualization of gender might entail (e.g., our theorizing on gender can be affected by who the primary childcare-giver is, if a family is comprised by a couple of lesbian women or homosexual men, etc.).

^4^While non-work identity salience could also be theoretically related to subsequent leadership status, within domain effects tend to be stronger and more consistent (e.g., work- or career-identity salience predict work- or career outcomes; Lobel and Clair, 1992). Furthermore, while work identity salience and leadership status undoubtedly have bidirectional effects, and related work on career identity argues that career identity change typically follows employment changes (e.g., Ibarra, 1999), work identity can also precede changes in career trajectories (see Sugiyama et al., 2022).

^5^While these moderators may also interact with each other (i.e., a more inclusive culture buffers employees from the negative effects of mega-threats; Leigh and Melwani, 2019, preserving the positive relation between work identity salience and leadership status), we focus here on their independent, individual effects as a first step.

^6^These effects may not only be limited to female leaders of organizations, because prominent examples and research suggest that the critical event of childbearing—when gaining a female child—can also motivate fathers to become more (socially) sustainability (e.g., venture capitalist and billionaire philanthropist John Doerr; Meyer, 2021; see also Cronqvist and Yu, 2017).
